# First sequencing of ancient coral skeletal proteins

**DOI:** 10.1038/s41598-020-75846-4

**Published:** 2020-11-10

**Authors:** Jeana L. Drake, Julian P. Whitelegge, David K. Jacobs

**Affiliations:** 1 Department of Ecology and Evolutionary Biology, University of California, Los Angeles, USA; 2 NPI-Semel Institute, University of California, Los Angeles, USA; 3 Department of Earth, Planetary, and Space Sciences, University of California, Los Angeles, USA; 4 Present Address: Department of Marine Biology, University of Haifa, Haifa, Israel

**Keywords:** Biochemistry, Evolution, Ocean sciences, Planetary science

## Abstract

Here we report the first recovery, sequencing, and identification of fossil biomineral proteins from a Pleistocene fossil invertebrate, the stony coral *Orbicella annularis*. This fossil retains total hydrolysable amino acids of a roughly similar composition to extracts from modern *O. annularis* skeletons, with the amino acid data rich in Asx (Asp + Asn) and Glx (Glu + Gln) typical of invertebrate skeletal proteins. It also retains several proteins, including a highly acidic protein, also known from modern coral skeletal proteomes that we sequenced by LC–MS/MS over multiple trials in the best-preserved fossil coral specimen. A combination of degradation or amino acid racemization inhibition of trypsin digestion appears to limit greater recovery. Nevertheless, our workflow determines optimal samples for effective sequencing of fossil coral proteins, allowing comparison of modern and fossil invertebrate protein sequences, and will likely lead to further improvements of the methods. Sequencing of endogenous organic molecules in fossil invertebrate biominerals provides an ancient record of composition, potentially clarifying evolutionary changes and biotic responses to paleoenvironments.

## Introduction

Endogenous organic molecules in fossil biominerals have the potential to provide records of past molecular composition with implications for evolutionary and environmental reconstruction. Fossil DNA has frequently been the target biomolecule in fossil samples and is useful for phylogenetic reconstructions of past organisms’ relationships to similar extinct and extant taxa and their responses to environmental change (review by^1^ e.g.,^2–4^). However, DNA’s preservation for successful sequencing is limited to the past several hundred thousand years for vertebrate taxa^5,6^ and less than 10,000 years for ancient invertebrate DNA^7^.

In contrast, proteins can persist in the fossil record for hundreds of thousands to tens of millions of years, and they can inform identification and significant phylogenetic reconstructions of extinct organisms, including for hadrosaurs (e.g.,^8–10^). Preservation of these biomolecules in vertebrate skeletons may in part be due to the embedding of cells in the biomineral. For instance, vertebrate bone, which contains osteoblasts, has a relatively high organic content (~ 20–30% by weight^11^).

Unfortunately, cells are not generally embedded in most invertebrate biominerals^12^; therefore, organic matter preservation for these taxa is minimal. However, invertebrate skeletons and shells retain specialized extracellular proteins associated with the biomineralization process^12^. Thus, these invertebrate biomineralization proteins provide a potential sequencing target. For corals, such proteins become embedded in individual skeleton crystals^13^, protecting them from degradation for long periods of time, and nitrogen likely derived from biomolecules in invertebrate biominerals has been analyzed for isotopic composition and presence of peptide bonds in invertebrate samples tens to hundreds of millions of years old (e.g.,^14–17^). For instance, δ^15^ N analyses of coral skeletons from the Triassic reveal that amino acids are preserved in fossil corals for hundreds of millions of years^15^, nearly as long as there have been reef-building scleractinian corals^18^. Yet, invertebrate biominerals contain much lower starting amounts of organic matter than their vertebrate counterparts, ~ 1% or less^19–22^, potentially leaving little sequenceable protein material intact. Until the present work, the oldest sequenced invertebrate “skeletome” data derive from ancient mollusk shells dating to several thousand years^23,24^, although there have been suggestions that somewhat intact proteins older than the Holocene do indeed remain in fossil invertebrate remains. For instance, glycoproteins extracted from an 80 myo Trigoniida bivalve mollusk shell were characterized as having ~ 5% sequence proportion of DYDY, which is nearly half such content found in a related extant molluscan taxon^19^, although the identities of the proteins were not determined.

The mechanisms of coral biomineralization have been intensively studied for over 150 years (review by^25^). Advances in the past decade include sequencing the skeletal proteomes of several modern taxa^26–29^, characterizing the carbonate chemistry conditions of the calcifying space that likely impact calcification in response to ocean conditions^30–32^, and developing coral cell cultures that precipitate aragonite at comparable rates to intact corals^26,33,34^. There has also been a general proliferation of sequenced coral genomes and transcriptomes (e.g.,^35–37^). Roles of several biomineralization proteins have been revealed including the ability of the highly acidic proteins called coral acid rich proteins (CARPs) or skeletal aspartic acid-rich proteins (SAARPs) to precipitate aragonite from seawater^38^, and the high enzyme activity of the coral skeletal carbonic anhydrase STPCA2^39,40^. However, the function of most of the approximately 100 known coral skeletal proteins remain to be established. Some protein roles may be suggested by their persistent interactions with the mineral once formed. For instance, proteins involved in nucleation of aragonite or in adhering amorphous calcium carbonate nanoparticles together toward their recrystallization to aragonite^13,41^, may be more tightly bound within the mineral and may resist degradation. In this context, fossil biomolecular data may help clarify roles of proteins currently of unknown function. Thus, important information may be forthcoming in addition to the utility of these protein sequences in organismal phylogenetic reconstruction.

To examine the persistence of coral skeletal proteins older than the Holocene epoch, we analyzed in-depth one modern and several Pleistocene Stage 5E Caribbean corals. As aragonite can recrystallize into secondary aragonite or calcite, with a potential loss or degradation of proteins in the process, we determined the samples’ mineral integrity by x-ray diffraction and inductively coupled plasma mass spectrometry analysis of element/calcium abundance. We then used racemization analysis of free and hydrolyzed amino acids and protein visualization to establish that any sequenced proteins are likely endogenous rather than modern contaminants. Finally, we sequenced extracted proteins using liquid chromatography with tandem mass spectrometry. This work yielded the oldest known invertebrate protein sequences and suggests that highly acidic proteins resist degradation through intimate interactions with the mineral phase and may be useful targets for further analysis within the invertebrate fossil record.

## Results

### Skeleton integrity and organic matter preservation

**Figure 1:**
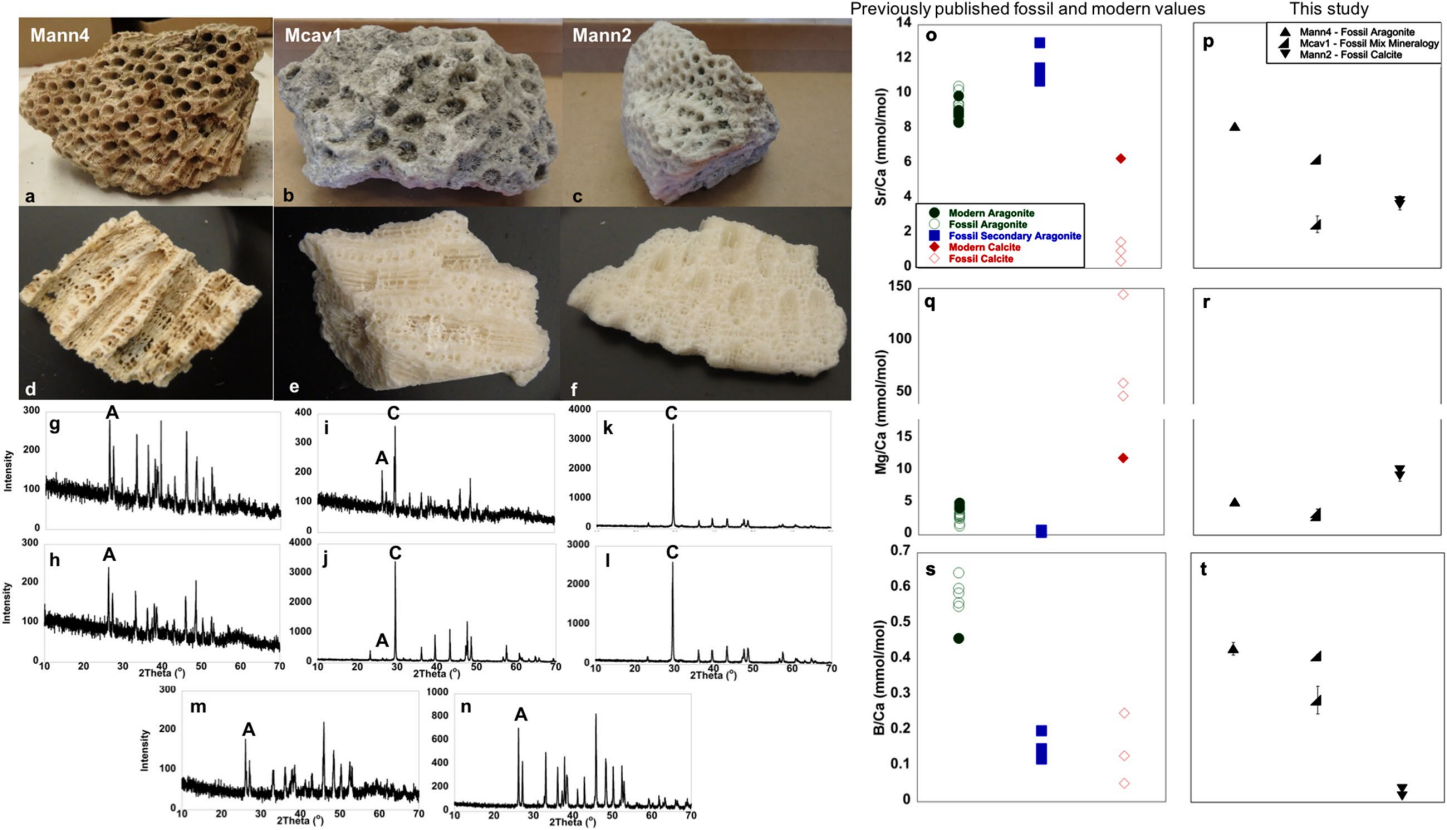
Fossil coral specimens obtained from the Natural History Museum of Los Angeles under various states of mineral preservation. One *Montastraea cavernosa* and two *O. annularis* specimens plus one modern *O. annularis* specimen were analyzed visually (**a**–**f**), by x-ray diffraction (**g**–**n**), and mass spectrometry (**o**–**t**). Characteristic aragonite peaks at 26 and 27 degrees 2ϑ are noted by ‘A’ and calcite peak at 29–30 degrees 2ϑ is noted by ‘C’ (**g**–**n**). *O annularis* 4 (Mann4; **a**, **d**, **g**, **h**) retains element/Ca signatures (**o**–**t**) suggesting that it remains > 90% primary aragonite. The modern *O. annularis* sample also remains 100% aragonite (**m**, **n**). The *M. cavernosa* (Mcav1; (**b**, **e**, **i**, **j**) has recrystallized by 15–30% to calcite and *O. annularis* (Mann2; **c**, **f**, **k**, **l**) has recrystallized entirely to calcite (**o**–**t**). Uncleaned *O. annularis* ‘Mann4′ (**a**), *M. cavernosa* ‘Mcav1′ (**b**), and *O. annularis* ‘Mann2′ (**c**) specimens. Clean *O. annularis* ‘Mann4′ (**d**), *M. cavernosa* ‘Mcav1′ (**e**), and *O. annularis* ‘Mann2′ (**f**) fragments showing degree of preservation of corallites and coenostea. XRD patterns for each specimen are beneath that specimen’s photographs; diffractograms for powders milled within corallites are images **g**, **i**, **k** while diffractograms for powders milled between corallites are images. XRD patterns for a modern *O. annularis* are shown for most recent growth (**m**) and older growth from ~ 10 cm deep (**n**). Element/Ca ratios for all fossil samples are shown in **p**, **r**, **t** to be compared with values shown in **o**, **q**, **s** from^94–102^ while such ratios for modern samples were determined from modern LANHM specimens for this study.

Five modern and three fossil coral specimens were interrogated for the quality of their mineral preservation (SI Table 1). Fossil corals that were collected in 1975 from exposed Key Largo Formation deposits, with youngest ages of 125 to 138 kiloanna (ka)^42,43^, and later donated to the Natural History Museum of Los Angeles County (NHMLA) were loaned to the authors. X-ray diffraction of these fossil specimens showed that *Orbicella anularis-*2 (hereafter: Mann2) has recrystallized to Mg-calcite and calcite, *Montastraea cavernosa-1* (hereafter: Mcav1) is 70–85% aragonite and 15–30% calcite, while *O. annularis-*4 (hereafter Mann4) is 93–100% aragonite and 0–7% calcite (Fig. 1a–l). A modern *O. annularis* specimen used for in-family comparison is 100% aragonite (Fig. 1m,n). Of the fossil corals, only Mann4 exhibited Mg/Ca, Sr/Ca, and B/Ca ratios within the range of modern and fossil primary aragonite (Fig. 1o–t). Together, our data suggest that the fossil specimen Mann4 remains mostly primary aragonite.

**Figure 2:**
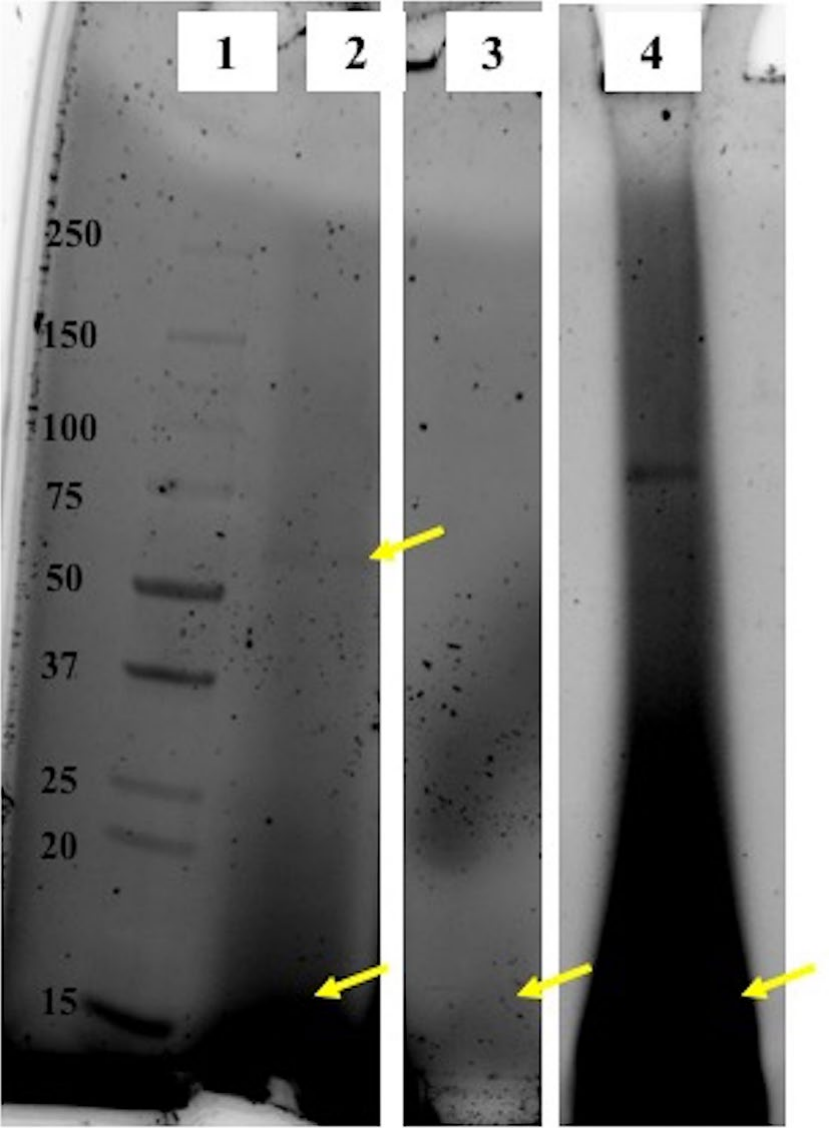
Fossil coral skeletal proteins separated by SDS-PAGE in order of least recrystallized to most recrystallized specimens. Lane 1 is the ladder, lane 2 is Mann4 acid insoluble matrix (AIM) pellet, lane 3 is Mcav1 AIM pellet, and lane 4 is Mann2 AIM pellet. Mann4 AIM pellet displays a standard acid-extracted biomineral protein smear with a band apparent at approximately 60 kDa (top yellow arrow). All specimens’ AIM pellets exhibit presence very small peptides (< 20 kDa) indicative of protein degradation (near lower yellow arrows), although Mann2 also retains a protein band at 75–80 kDa. Samples and ladder of Mann4 and Mcav1 were run on the same gel which has been cropped to show only these lanes, whereas Mann2 was run on a separate gel. Brightness and contrast of all gels have been adjusted. The uncropped gel images, both adjusted and unadjusted are provided in SI Fig. 1.

SDS-PAGE indicated that proteins were among the biomolecules extracted by acid hydrolysis of cleaned fossil skeleton powder for all fossil specimens (Fig. 2). However, these proteins were degraded as evidenced by a bias toward small peptides (i.e., in the low molecular weight area of the gel) in all specimens. Further, fossil corals Mcav1 (70–85% aragonite), Mann2 (fully recrystallized to calcite), and Mann4 (> 90% primary aragonite) exhibited total hydrolysable amino acid (THAA) D/L Asx (aspartic acid plus asparagine) values of 0.634, 0.611, and 0.380, respectively (Table 1). For comparison, THAA D/L Asx of a modern *O. annularis* skeleton was 0.212.

**Table 1:** Average total hydrolysable amino acid (THAA) relative percent, D/L Asx and Glx, and % FAA Asx of proteins extracted from freshly collected, cored, and museum- and privately-held coral skeletons.

Amino acid	Modern *Porites australiensis* (63)	Modern *Acropora palmate* (61)	Pleistocene *Acropora palmate* (61)	This study						
				Modern *Fungia* sp.	Modern *Pocillopora damicornis* and *P. acuta*	Modern *Porites lobata*	Modern *Orbicella annularis* -aragonite	Pleistocene *Orbicella annularis* -aragonite (“Mann4”)	Pleistocene *Orbicella annularis* –mixed mineralogy (“Mcav1”)	Pleistocene *Orbicella annularis* -calcite (“Mann2”)
%Asx	60.3	52	47	51.9	36.8	51.5	56.3	36.9	37.6	45.0
%Glx	13.9	19	20	15.8	19.0	14.6	18.3	18.2	16.4	13.1
%Ser	8.9	8	1	10.5	8.5	5.2	5.2	4.2	2.7	5.4
%Ala	5.1	5	14	6.3	11.1	8.0	6.5	16.0	15.6	11.9
%Val	3.7	5	7	6.1	8.5	5.6	5.8	8.2	7.5	7.5
%Phe	2.5	2	2	3.5	7.1	7.1	1.7	2.7	11.6	1.6
%Ile	1.9	2	3	2.9	3.9	3.4	3.2	9.4	4.1	4.1
%Leu	3.7	6	6	3.0	5.1	4.6	3.2	4.3	4.4	4.4
THAA D/L Asx	0.173	0.181	0.821	0.131	0.141	0.134	0.212	0.38	0.634	0.611
THAA D/L Glx	–	–	0.793	0.072	0.072	0.109	0.107	0.279	0.547	0.470
%FAA Asx	–	63	54	4.4	11.0	15.4	2.2	33.7	38.9	35.0

Asparagine is converted to aspartate during hydrolysis of proteins/peptides, and the two together are reported as Asx.

### Fossil and modern coral skeletal protein sequencing

No coral proteins were sequenced from cleaned skeleton powder of Mann2 (fully recrystallized to calcite) or Mcav1 (75–80% calcite). In contrast, Mann4 (> 90% primary aragonite) yielded six coral proteins within our stringent criteria containing peptides that were sequenced by LC–MS/MS after trypsin or trypsin-then-GluC digestion; all proteins had either more than one peptide detected at least once, or one peptide detected multiple times (Table 2 and SI Table 2). Five proteins were detected in the acid-insoluble fraction and one was detected in the soluble fraction, with no overlap between the two fractions. This is contrasted by the 61 proteins detected across all solubility and digestion fractions of proteins extracted from the newest growth of a modern *O. annularis* (SI Table 3). Proteins sequenced from fossil Mann4 skeleton include one of the highly acidic skeletal proteins known as acidic skeletal organic matrix protein (acidic SOMP)^27^ or SAARP3^28^ in *Acropora* spp. and P27 in *Stylophora pistillata*^26^, and also detected here in our modern *O. annularis* (SI Table 3). Although the Blast2GO annotation calls this protein acidic SOMP-like, we use SAARP3 here to be consistent with the most recently published terminology^28^ and because the gene is clearly a sub-group within the CARPs4/5 or SAARPs1-3 gene family (SI Fig. 2). Other fossil proteins sequenced include a coadhesin which was also detected in our modern *O. annularis*, a lanC-like protein, a polyamine-modulated factor-binding protein, and two uncharacterized proteins.

**Table 2:** Six proteins detected in a well-preserved Pleistocene *Orbicella annularis* (Mann4).

Database	Accession	Score	Mass	No. of matches	No. of significant matches	No. of sequences	No. of significant sequences	Blast2GO description	e-value	%Coverage fraction	Digestion
Orbicella_Annularis	g39268.t1	71	112,411	3	2	3	2	Uncharacterized protein LOC110058287 isoform X2 [Orbicella faveolata]	0	2 AIM	Trypsin1
Montastraea_cav	Montastraea_cavernosa_96538	60	135,355	2	2	2	2	Coadhesin-like, partial [Orbicella faveolata]	0	1 AIM	Trypsin1
Platygyra_carnosus	Platygyra_carnosus_37674	45	22,588	8	5	1	1	Acidic skeletal organic matrix protein-like [Orbicella faveolata]	8.12E−95	8 AIM	Trypsin1
Orbicella_Annularis	g29668.t1	82	38,126	12	7	1	1	lanC-like protein 3 isoform X	0	3 AIM	Trypsin2
Montastraea_cav	Montastraea_cavernosa_28848	38	21,179	3	3	1	1	Polyamine-modulated factor 1-binding protein 1-like [Orbicella faveolata]	1.50E−93	3 AIM	GluC2
Platygyra_carnosus	Platygyra_carnosus_62468	37	18,905	2	2	1	1	–-NA–-	No blast hit	4 AIM	Trypsin1

## Discussion

Sequencing of six fossil coral proteins (Table 2), three of which are also found in modern coral skeleton (^26,27^, present study), provides further confirmation that coral skeletal proteins are specific components of the organic matrix embedded within individual aragonite crystals rather than simply cellular contamination^13,44^, as peripheral proteins in the fossil samples would be accessible to degradation processes over the past ~ 100 ka. In the case of SAARP3, strong interactions likely persist between the acidic domains of the protein and calcium atoms in the biomineral^13^. This protein is a member of the CARPs4/5 or SAARPs1-3 family (SI Fig. 2), one member of which has been shown to lead to the precipitation of calcium carbonate from unamended seawater^38^. Further, blasting indicates that the peptide detected in this protein does not have a BLAST hit in *Homo sapiens*, so it is not a contaminant of the extraction and sequencing. Having shown that the proteins sequenced from the fossil specimen are not likely contaminants (SI Tables 4 and 5), we can focus on the remaining fossil proteins. Like SAARP3, coadhesin has been sequenced previously from modern coral skeleton^26–28^. Coadhesin may persist in a sequenceable form in fossil corals due to its relatively high abundance, as suggested in modern skeleton (SI Table 3). Coadhesin, a transmembrane protein, contains multiple extracellular thrombospondin type-1 repeats, possibly allowing this protein to serve a role in adhesion of calicoblastic cells to organic matrix on and in the skeleton^27^. Further, thrombospondins may form functional triple helices similar to collagen and peroxidasin^45^ two proteins known from coral skeleton^26,27^. They have been implicated in structuring the other framework proteins of the ECM in mammalian osteoblasts^46^ and inhibition of osteoblast differentiation^47^. This structural set-up of the coral ECM is engulfed by the growing mineral where it is preserved for, potentially, 100s ka. Two other proteins, a polyamine-modulated factor-binding protein that may play an adhesion and scaffolding role in the biomineralization process, as suggested by the protein’s adhesive properties in sperm^48^, and a lanC-like protein which may associate with the cell membrane^49^, were also detected in the fossil *O. annularis* skeleton but not in the modern one. One uncharacterized protein is similar to a protein previously sequenced from *S. pistillata* (P16^26^) while the other is unique to *O. annularis* skeleton among all coral skeletal proteomes, although orthologs are present in genomes and transcriptomes across many scleractinian taxa (reefgenomics.org, NCBI). In general, annotation of several of the detected fossil proteins suggests that they were likely involved in adhering coral cells to the growing skeleton and in structuring the physical environment of the calcifying space, bringing them into direct contact with the mineral.

While 61 proteins were sequenced from the multiple fractions of modern *O. annularis* skeleton, only three of these, and six proteins total across all digestion fractions, were detected in fossil skeleton. This is likely due to two issues. As analysis by SDS-PAGE shows, fossil proteins had likely degraded into smaller peptides (Fig. 2). Further, racemization of Arg and Lys would minimize the efficacy of trypsin^50–52^, although the stochastic nature of racemization means that, even after 100s ka, at least half of the enzyme target amino acids should be in a cleavable form. We attempted to overcome diminished effectiveness of tryptic digestion on fossil samples by further digesting the post-trypsin peptides with GluC, which selectively cleaves after Asp or Glu. Increased protein sequencing has been observed when using multiple enzymes on modern specimens^53,54^, and we detected two additional proteins above our cutoff settings in post-GluC digestions of fossil *O. annularis* acid-insoluble skeletal protein. Coral skeleton is notoriously low in organic matter abundance with estimates of skeletal organic matter content as low as 0.01%^22^ and as high as several percent^55^, so that the combined effects of low starting material, protein degradation, and racemization of enzymatic digestion targets places severe limits on the sequenceability of fossil coral proteins that would be encountered. Skeletons of invertebrates with comparable amounts of starting organic matter content such as mollusk shells^19^ and echinoderm tests and spines^20,21^ at 0.3–4% and ~ 0.1% by weight, respectively, may face this issue as well.

In work of this nature, care must be taken to ensure that reported proteins are not modern contaminants^56^; well-preserved specimens must be chosen and best practices in handling samples must be employed^57^. Our crystallographic and trace element analyses allowed us to select a fossil coral specimen that retained its primary aragonite mineralogy (Fig. 1). Further, in addition to extensively cleaning all skeleton powders, we handled fossil and modern powders separately in age specific glove bags and months apart, with fossil *O. annularis* skeletons handled first. We also examined biochemical signatures of age and persistence of intact proteins.

Amino acid racemization has been used for the past 50 years to study fossil samples ranging in age from 500 to 300,000 years old^58–60^. In the present study, Mann4 exhibited THAA D/L Asx lower than similarly-aged Atlantic and Caribbean Pleistocene-aged corals^61,62^. This may be due to the fact that most of the Mann4 THAA Asx was made up of polymerized amino acids (33.7% FAA) whereas FAA accounted for most of the coral THAA Asx in^61^ (~ 60% FAA based on their Supplementary Figure EA 1; see our Supplementary document for details on quantitation), with the FAA pool being drawn from hydrolysis of terminal amino acids in degraded proteins for which racemization had already occurred^63–65^. In contrast to Mann4, the higher D/L Asx in Mann2 and Mcav1 may be due to degradation of proteins, potentially linked with recrystallization, as observed in protein extracted from the partially recrystallized Mcav1 and analyzed by SDS-PAGE (Fig. 2). This is similar to the process by which Asx racemization is greater when demineralization of bone results in degradation of the collagen triple helix^66,67^. Differences in protein content between the fossil coral specimens is likely not due to differences in collection or preservation as documentation for the fossil skeletons indicates that they were collected by the same person at the same time and then stored together in warehouses at the NHMLA after their donation.

All three Pleistocene corals also exhibited relative amino acid molar concentrations similar to archived modern specimens reported here as well as archived material from a modern *Porites* skeleton and modern and fossil *Acropora* skeletons for which amino acid relative quantification was performed as part of racemization analysis^61,63^ (Table 1). Even Mann2, fully recrystallized to calcite, retained a THAA relative composition roughly similar to its modern counterparts with a persistent bias toward the acidic residue-containing amino acid groups, Asx and Glx. However, compared with modern coral skeletons, we observed decreased relative Asx and Ser and increased relative Val in Mann4 and Mcav1, decreased relative Glx in Mann2, and increased relative Ala in all three fossil specimens (Table 1, SI Table 6). The difference in amino acid composition could be due to loss of highly acidic proteins, of which there are multiple types in coral skeleton^13,41,68^, and which would reduce the remaining Asx pool while increasing the relative abundance of the remaining non-acidic residues. We also observed a bias toward smaller peptides observed by SDS-PAGE (Fig. 2). Together with the non-modern D/L Asx and D/L Glx values, these are indicative of both very old proteins and of protein degradation and loss in the fossil coral skeletons. Further confirmation that we did indeed sequence fossil coral skeletons is provided by degradation signatures in detected peptides. In particular, the detected peptide in SAARP3 in the acid-insoluble SOM fraction is suggested by MS1 data to be deamidated in both asparagines in the peptide. This deamidation is a known degradation feature observed in other paleoproteomic analyses (e.g.^69,70^). Further effects of proteins being locked in aragonite crystals may include differential production of isoaspartate (or gamma glutamic acid) during asparagine (or glutamine) deamidation^71,72^; unfortunately, the low peptide yield was insufficient to pursue this analysis in the present study. Finally, our phylogenetic analysis shows that SAARP3 is a coral-specific protein (SI Fig. 2). In sum, we are confident that the six proteins sequenced from the fossil *O. annularis*, Mann4, are coral proteins of the same age as the skeleton.

It should be noted that extraction of coral proteins for sequencing is destructive. While owners of gifted or loaned coral skeletons were informed of this ahead of time and approved such use of the specimens, we remain cognizant that our use of portions of the samples precludes analysis of these portions in the future. We therefore sought to minimize the amount of material used for sequencing. Some modern coral skeletal proteome sequencing has used 10–30 g of cleaned skeleton powder^27,28^, but comparable protein detection can be obtained from approximately 1 g of cleaned modern material^29^. In this study, we found that this smaller amount of skeleton allowed sequencing of some fossil proteins and reinforces that these biomolecules are potentially available for sequencing from invertebrate biominerals aged over 100 ka. More material may yield better detection, and hence more sequenced peptides, in future studies but specimens should be carefully chosen to minimize loss of irreplaceable samples. Further, enzymatic digestion success is likely minimized by amino acid racemization. While we show here that we can successfully sequence several known modern skeletal proteins in fossil coral specimens, future method refinement may consider inclusion of other, non-enzymatic, peptide cleavage methods; this could include the use of cyanogen bromide (e.g.^73^), although significant accumulation of methionine oxidation in fossil specimens would minimize its effectiveness^74^.

In summary, we show that fossil coral skeletons that retain their primary aragonite mineralogy preserve endogenous proteins that we extracted and sequenced by standard methods, while proteins in recrystallized fossil coral skeleton are too degraded for sequencing. To be retained in the skeleton for over 100 ka, these proteins must have been intimately associated with the aragonite crystal. Our work supports this and pushes back the age at which these phylogenetically informative skeleton biomolecules can be obtained from the invertebrate fossil record.

## Methods

### Sample description

Colonies of fossil *Orbicella annularis* and *Montastraea cavernosa* skeletons were borrowed from the Natural History Museum of Los Angeles (NHMLA) Invertebrate Paleontology Department (Fig. 1). All specimens were originally collected from Pleistocene deposits in the Key Largo Formation (FL) aged 125 to 138 ka^42,75^. These and modern corals of several species, also NHMLA collections, and the surface layer of a privately-owned *O. annularis* were analyzed for skeleton integrity (SI Table 1).

### Sample cleaning

Slabbed coral fragments were soaked in equal parts 30% hydrogen peroxide and 3% sodium hypochlorite after Stoll^76^ and then ground to 125 µm . Skeleton powder was cleaned three additional times before being dried at 40 °C. Cleaning was sufficient to remove contaminant proteins, as determined from phosphate buffered saline solutions soaked on cleaned skeleton powders and then concentrated on 3 kDa Amicon Ultra centrifugal filter units (Millipore), at the detection level of bichronoic acid assays (SI Table 4), of Stain-Free SDS-PAGE (Bio-Rad) and imaging (SI Fig. 3), and were at concentrations three orders of magnitude lower than that observed in cleaned coral skeleton powder by amino acid analysis^61^. All clean powder was only handled in age-specific glove bags (i.e. separate bags for fossil and modern), and modern samples were never handled at the same time as fossil samples for any biochemical analysis.

### Skeleton integrity

Duplicate milled sub-samples of cleaned skeleton were cleaned after Stoll^76^ and dried at 60 °C before analysis on a Panalytical X’Pert Pro X-ray Powder Diffractometer (Malvern) on a zero diffraction background plate. Spectra were analyzed in X’Pert Hi-Score software and relative amounts of aragonite and calcite were determined by the Reference Intensity Ratio method^77^ using 01–072-1652 calcite and 00–041-1475 aragonite references for all samples. After x ray diffraction analysis, elemental composition of powders was measured on an Element XR HR-ICP-MS (Thermo Fisher) using *Cibicides wuellerstorfi* (CAM-wuell) as a consistency standard^78^.

### Amino acid racemization

Amino acids for racemization analysis, both free and total hydrolysable amino acids, were extracted, hydrolyzed, and evaporated to dryness from cleaned skeleton powders by standard methods^79^. All samples were prepared in duplicate and analyzed at the Northern Arizona University Amino Acid Geochronology Laboratory using standard methods with modifications for microfossils^80–82^. Rehydrated samples were spiked with L-homo-arginine as an internal standard and then injected into an HPLC fitted with a reverse-phase C18-packed column. ‘Blank’ samples were included.

### Protein extraction

Approximately 1 g cleaned powder from each coral was decalcified in 0.5 M glacial acetic acid. Acid insoluble matrix (AIM) pellets were rinsed twice in ice-cold 80% acetone whereas acid soluble matrix (ASM) was precipitated in ice-cold 100% acetone and then rinsed twice in ice-cold 80% acetone. Pellets were immediately submitted for protein sequencing. A subset of extracted proteins were separated by SDS-PAGE on 4–20% Mini-PROTEAN TGX Stain-Free™ precast gels (Bio-Rad) which were imaged on a Bio-Rad ChemiDoc XRS + imager following UV light exposure for 5 min.

### LC–MS/MS protein sequencing

Skeletal protein AIM and ASM samples were dissolved in 2% SDS buffer and digested using either a filter aided sample preparation (FASP; Mann4)^83^ or a multi-enzyme digestion filter aided sample preparation protocol (MED-FASP; Mcav1, Mann2, Mann4, and the surface layer of a modern *O. annularis*) modified from^54^. Briefly, protein was dissolved in SDS buffer and placed in a 30 kDa Microcon Centrifugal Unit (Sigma Aldrich), SDS was displaced using an 8 M urea solution and then the sample was diluted to 2 M urea and digested with trypsin (Promega). Digested peptides were moved through the filter into a micro-centrifuge tube (low retention; Fisher). Any undigested material that remained on the filter was then digested with Glu-C (Promega) and peptides centrifuged into a second micro-centrifuge tube. Each fraction was analyzed separately on an nano-liquid-chromatography system coupled to a benchtop high-resolution orbitrap mass spectrometer (QE-Plus; Thermo Fisher) and operated in positive ion mode with data-dependent acquisition. MS1 was performed at resolution of 70,000 (at 400 m/z) and MS2 at 17,500. Peak lists were extracted from raw spectra and processed using a Mascot (2.4; Matrix Science) server against *Montastraea cavernosa*, *M. faveolata*, and *Platygyra carnosus* protein databases downloaded from comparative.reefgenomics.org^35^, an *O. faveolata* protein database^84^, and the *O. annularis* genome predicted protein database^85^ under NCBI BioProject 550266. A common contaminants database downloaded from the Max Planck Institute of Biochemistry, Martinsried, and a UniProt-Human database were included in the analysis to test for contaminants. We also separately ran the LC–MS/MS data in Mascot against UniProt-bacteria, UniProt-cyanobacteria, and UniProt-fungi databases and confirmed that all detected peptides from those databases did not match peptides assigned to corals (SI Table 5). We also ran LC–MS/MS data for a preparation blank sample against the coral, common contaminants, and UniProt-Human databases to confirm that other contaminant from the preparation process were not assigned to corals (SI Table 5). For all Mascot runs, we applied carbamidomethylation of cysteine as a fixed modification and oxidation of methionine, acetylation, and deamidation of asparagine and glutamine as variable modifications. Enzyme specificity was set to trypsin with one missed cleavage allowed. Mass tolerances were set to 10 ppm and 20 mmu for precursor and product ions, respectively, and precursor charge was set to 2 + , 3 + , or 4 + .

Initial decoy searches were performed in Mascot using a 1% false discovery rate to determine the appropriate significance value setting. Next, we performed Mascot error-tolerant searches with this significance setting. Only protein sequences above the cutoff score with at least two independent significant peptides detected, or one peptide detected significantly multiple times, were retained. We blasted these sequences against the NCBI nr database in Blast2GO. Further, we BLASTed returned proteins against the NCBI *Homo sapiens* database and manually checked hits with high sequence similarity for identity of LC–MS/MS detected peptides; if ‘coral’ and human peptides were identical, we manually removed the protein sequence from our list of coral skeletal proteins. The mass spectrometry proteomics data have been deposited to the ProteomeXchange Consortium via the PRIDE^86^ partner repository with the dataset identifiers found in SI Table 7.

### Phylogenetic analysis of SAARP3

CARP4/SAARP1, CARP5/SAARP2, and P27/acidic SOMP/SAARP3, previously sequenced from coral skeletons^26–29^, were blasted against the cnidarian predicted protein databases in comparative.reefgenomics.org^35^. They were also blasted against NCBI and the top non-cnidarian hits with E-values better than e-20, two *Crassostrea gigas* sequences*,* were retained. Multiple sequence alignments were generated in T-Coffee^87,88^. Aligned protein sequences were trimmed using the TrimAl v1.3 alignment utility in Phylemon2 using the gappyout method^89,90^. The most appropriate model was chosen in ProtTest 3^91^, and then maximum likelihood trees were constructed in PhyML using the WAG + G + I substitution model with bootstrap set to 1000 and all other pre-set parameters^92^.

### Amino acid composition statistics

Relative content of each amino acid (THAA) was compared between the replicate data for each fossil coral skeleton reported on here versus average values from modern *O. annularis, Fungia* sp. *Pocillopora damicornis, P. acuta*, and *Porites lobata*, plus previously reported values from modern *Acropora palmata*^61^ and *Porites australiensis*^63^, as reported or reproduced in Table 1, in RStudio^93^. Shapiro–Wilk normality tests showed that all modern amino acids exhibited normal distribution, so that Student’s t-tests were applied (SI Table 6).

## Supplementary information

Supplementary Information 1.

Supplementary Information 2.

Supplementary Information 3.

Supplementary Information 4.

Supplementary Information 5.

Supplementary Information 6.

Supplementary Information 7.

Supplementary Information 8.

## Data Availability

The mass spectrometry proteomics data have been deposited to the ProteomeXchange Consortium via the PRIDE^86^ partner repository with the dataset identifiers found in SI Table 7.
