# Supplementary material for: First sequencing of ancient coral skeletal proteins
Source: Sci Rep. 2020 Nov 10;10:19407. doi: 10.1038/s41598-020-75846-4 (PMC7655939; doi:10.1038/s41598-020-75846-4)
Supplement: Supplementary file 2 — Supplementary Information 2. [file 41598_2020_75846_MOESM2_ESM.rtf]

>g39268.t1_Insol_FASP_trypsin1VDHICLTKFDRDQKTALMIPAKKAFGDVDDWDEKVLGRLCNLLEALPVRDILKLASDVVTKAIDSLVKNDFTIPQAKAIIAKLKEQWKEVKTWSAPQLQKVGKLLKDFSVEDLKYLSKEQFQAIADVVSKLKLDAGQLRVLAAKAKELLGSPDKWNKDNIKELGNIVAGLLPSELKQIGEQVIKDSLQALKEVDFSLDQAQEIVEKLKSSIDLSQLKKEDVIALAKSIDGFLSSDVGKMAQAAVFAAFPEMKIAKDVAIPVLRQFIKKYEENPSAGKNIAQLGEFAVALSRGEVNAENVDDVIANLDQLGLIPWDKTQVLTLAKKISTKWGDFNTTDADDSDSPNWGFLNMKKLGRIVLGIAKEQLRDLPIRGIEDVIDVLGREKDWDRGQIVMVLTRLREYWEMENLDFSNFTEVDIDSLGTFLRGLAQDELKKLPEKILLTAIRRLGEETGLPEDKLKAMAYLAVELFKNQTGVDILNSSHIEDLGSLVAGLDRKTLRKIAKDAFIDNLYNIARAKGYDVKKLEEIAKLAKQHFDKSDVAEWIGDEWRDLGPAALGLDPSDLERINLDSLEEMLDEFGTFNFSKSQANSLVEAAKKAWDESDAGKWTGEKLRQLGSLVKGLDTTDIKKLGKEAFEEAVGVWGKYLDVDMETLEALAEKAKEYLTNGDISKLTAQLAKRIGRVVLGLTPDDLDKLKLDNIDMIAALGKWKEWSNDQLDRLKPKVREFLKQNKDDDAFMSLGQLALSLTKDDIAKMSQKAFRLAVKQLSEIEGWSDEQLKAIIAKAKTVWAQAANQWDKDQVSELGKTLKALSTADIPKLKTKVVDVIPPEVIEDMSVSQLQVENVYYADTRVNVIHRASVRLCMNSVATGQEMVREKQILQDQGKAFSADQYKAMQAPQVQAISSAKKGSLSMAQQAAIKSVIDSDPDEEDPWGPDEDDDDDDCSAAIFNNDNDNDNDNDNDSDSDSGSDSGSDSDSDNDNDRNTCSSSSHVTVTFTSMMVAPVGMLMR>Montastraea_cavernosa_96538_Insol_FASP_trypsin1FDQHRQNYKSAEDYRSTNYSSCEQEAKFTESKMPLLNWSWQLVLLLSILSSNIRSTVSQGCTSLVLNNNVKFSASSSVLGPGKPVVGGNDVWCAGIPTRNQWLKVDLGFRLLFDRVFVQGKARSNRSVAEYYLKTSNDDDVYVTITDPSVDRIRKFNGPLFNGDDVVVQNLSTPVEARYVIFNPREPSNILENYMCMRIDILSCKNVPPAIDGGWTDWSDWTPCLPCTHAVKTRSRTCTDPAPAFGGNQCSGHSEMNQLCPNNCVVPVDGNWSDWGAWSPCSETCGNGTAYRSRECNNPLPENNGKDCEGPAIDSKECFVQHCPVPGNWSSWGDWSPCSKTCGNGTITRTRTCDNPAPAYGGTDCVGAANMTKDCLDQLCPVPTPSPTPSMTSSVTPTSSVTVQPPVVDGGWSDWIYSPCDAQCGPGKQNRTRTCDNPLPSGGGADCQGPALETVDCNNGPCVDPNMPNIDLAFAIGATSANSNQSYALMKNTIKQFIDKYGVDNVHYSLIVYGDSVIRFVNFNNTFPPSASDLKAAIDAQPPVSGGPVLKDALQEGFRVFNESEGRPDAKMVLVVMTDENSGVDSNSLSTAVKPVEDLGVLVLSVGAGSTVDRNELSVISPNPKDVLSPTLGENPSVLAERIMERILRRNIPLIDVGFGIRASEINSSAIFTVMTETRDTINHRYGPGQVQYSVTVYGSKVTTNFNFDLDNALNLNALIAATNQLLDVPGPVDLPQVLTDAERLFQSHPNRRPFSERVFIAITDTAKSDNDSALISAGDALRRQGILVFSVNNTGDGLNAVTITQIDFLGFPTFTTVRSVVIAETIIKKALEVNMPLIDLTFAISATSISSDRTFLLMQTAINNIVSDYDIFRIHHSVIVFGSVATTPIDFGSNIPDKATLIRLVARLTEISGDVDLVLALEEAKKVYQLQEVRPDARRFLVVIMDNQSVNNVDDVNRAVTDLDNQDVVVIGVSVGNSTDPTDFEIITKDPRHIITVGVNKSPVELAKEIIDAIFISIRTAGFSQWAEWSACTKPCRSGGVAGTQRRTRVCVKSRLGCIGATVETRECNTEDCLGCEERGPLSDNAYLASSAVQPASWARLNTSNPGASQRAWCASVDDLENGGAYVQIDLGENVDVFRIATRGQDLAGQQRWVTSFFISNSSNGLSFDVFQEGGQRKDFDGNTDPGSIVFTDINITTRFIRFHPLNFSEQPCMQASVFGCTKS>Platygyra_carnosus_37674_Insol_FASP_trypsin1DNSSTYQGIPVKNVNLSVFLAGPRATLDLMVMVFLGAGNVEFGNETFRVQSGTLKFNIKISDWQFCDQGNETQCISRSNQNEIGQYLDLQLSIKSSAEPEEVEEEERQGSNKEAICVDDDPNEPDDDCPKIYNMGGKAEMVLNKGVLTGNNEYVAFPPGFPKFVSTGMMKSFSFRIPKFNNTCIIDPSVNLEAPIRNQPGGGA>Platygyra_carnosus_62468_Sol_FASP_trypsin1RLASNRAGQQAMALSALSANGWRKEPESLCSRISETEATAHRLQEELSTILRGLAALKEQVRDADDLAGDTETEKIRQEVDDVLRRQGTVLEEMVKSAESFPRRWAQFELNISQRKSLLLSIKAEERGIKQSDEMCPYRCETYSDRCRRHRRDQNLTELLWX>g29668.t1_Insol_FASP_trypsin2MFRSRSRCFVNGYEKFARSLGSVEYLRDLVQRVLQLIEERSPPHRSSCDGGLYVGAPGIGYAFYSVAESSEFSNIREQCLGKALEYMQVSLREVSRTPPNDDGIGASFLLGHAGIYAVSALVFNALGHQQETQQCIQKFLEMGNICRPVNFFRPGSDELFVGRAGYLCGSLLLNRKLGGNVVPSGVTRSVFDAIIESGRQYSQKHHSKSPLMYSYYRTEYLGAGHGLSSILQILLNFPEHFTDRKEVETLIQHAVDFVLSCEWPNGNYPPVPGEARDQEDELVHWCHGAPGVVYLLAKAYLTWKDDKYLQAAKRCAELTWQKGLLRKGPGICHGVAG>Montastraea_cavernosa_28848_Insol_FASP_GluC2AKTQEFEEGKTVLKLKENCLSKVETELAETKESYEKLMQERLSEYELEKDSMMKEINGLRCLQGLPTLKLVSNRESVTPRQEEKDGLVSAEKRTRNRKNNKLNSELTRAKEQLVRLKAELTMSNMQTRNLGTQLSSLREDSTKLEAELSTVRVFPKNSGQRRNSFSCYEETVRLEIELAEAK
