# Supplementary material for: First sequencing of ancient coral skeletal proteins
Source: Sci Rep. 2020 Nov 10;10:19407. doi: 10.1038/s41598-020-75846-4 (PMC7655939; doi:10.1038/s41598-020-75846-4)
Supplement: Supplementary file 8 — Supplementary Information 8. [file 41598_2020_75846_MOESM8_ESM.pdf]

Supplementary Information: First sequencing of ancient coral skeletal proteins


**Authors**

Jeana L. Drake[1,2*,3*+], Julian P. Whitelegge[4], David K. Jacobs[1,3+]

**Author Affiliations**

[1]Department of Ecology and Evolutionary Biology, University of California, Los Angeles, U.S.A.
[2]Department of Marine Biology, University of Haifa, Haifa, Israel
[3]Department of Earth, Planetary, and Space Sciences, University of California, Los Angeles, U.S.A.
[4]NPI-Semel Institute, University of California, Los Angeles, U.S.A.
[*]Present affiliations
[+]Corresponding authors: jeanadrake@g.ucla.edu, djacobs@g.ucla.edu


**Quantitation of Tomiak et al. 2016 %FAA**

As detailed in the Methods of [Tomiak, 2016], a fossil *Acropora palmata* skeleton was powdered, bleached, and then soaked in water at 140ºC for 6- or 24-hours to test for leaching of organic matter from the mineral phase. The leachate and decalcified skeleton were then interrogated for THAA and FAA content. Skeletal THAA and FAA are reported by those authors in their Supplementary Figure EA 1. For our comparison of %FAA content, we imported screenshots of the insets of that figure containing the skeletal AA data into Engauge Digitizer [Mitchell], extracted the total pmol/mg THAA and FAA for the 24-hour data, and calculated %FAA.

**SI Figures**

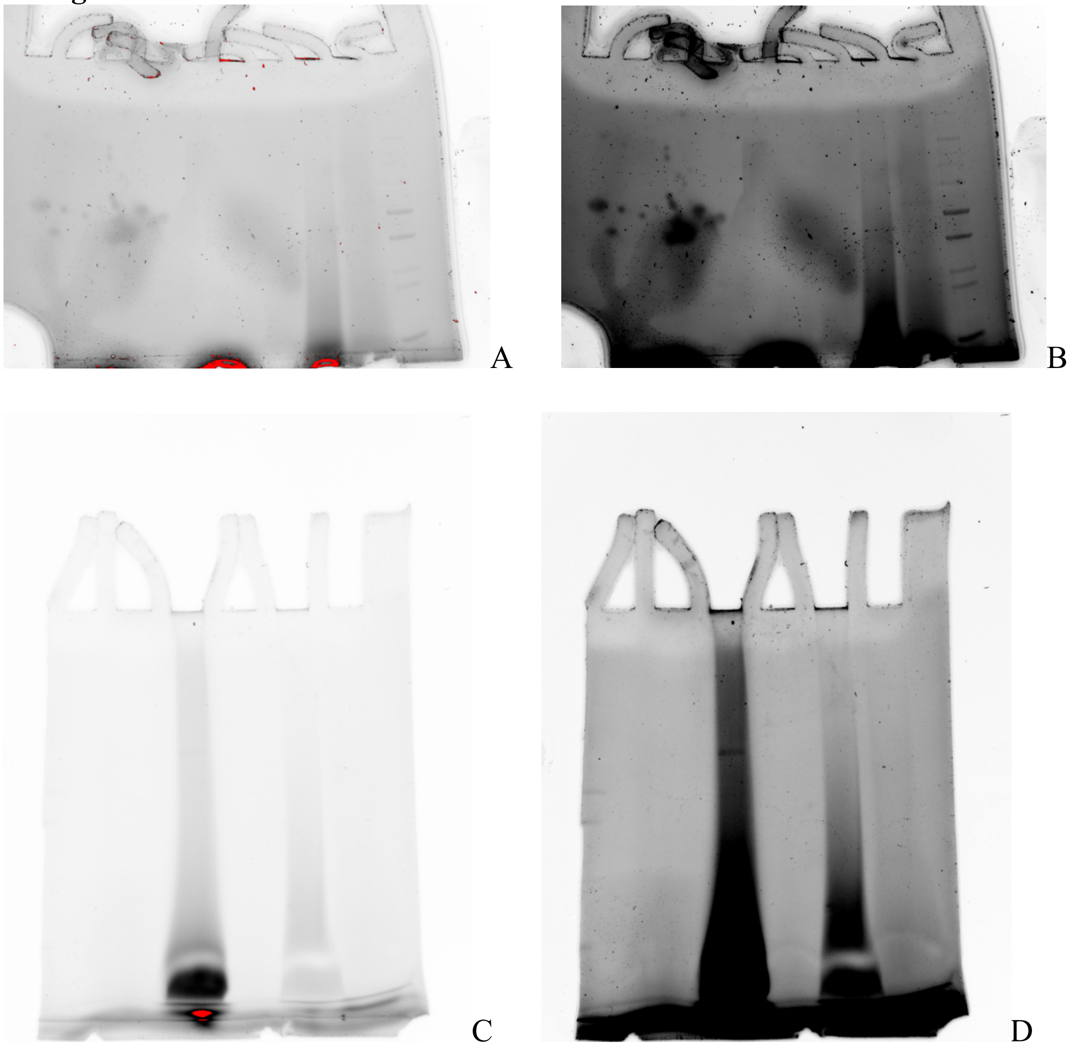


SI Figure 1. Full SDS-PAGE image of extracted fossil coral proteins rotated but otherwise unmodified (A, C) and with the brightness and contrast adjusted to better see smearing and banding (B, D). (B and D) were flipped or rotated and cropped to produce Figure 2 in the main text. Starting from the <u>right-most</u> lane in both images, the cropped lanes used in Figure 2 are the first, second, and fourth lanes of A and B, and the third lane of C and D. Molecular weight markers in C and D are in the sixth lane from the right (or left-most lane), with the 37 and 50 kDa bands just visible at the edge of the cut gel in D.

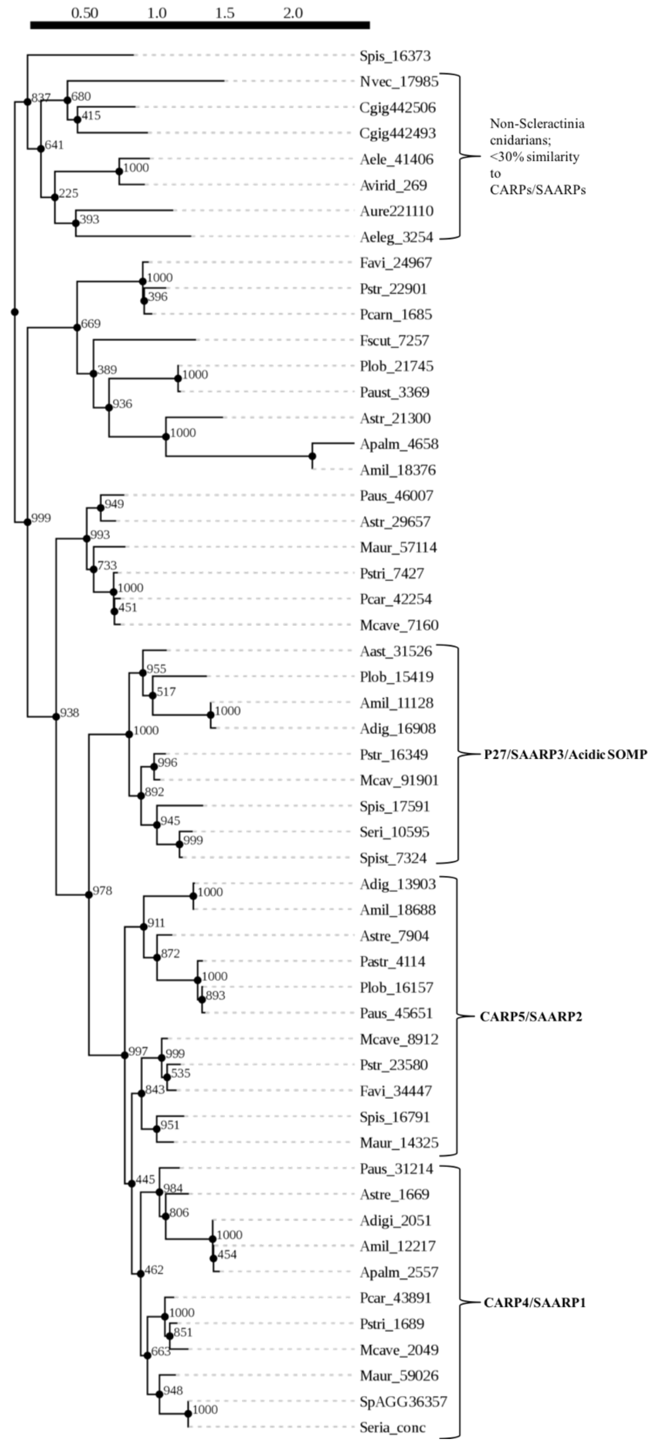


SI Figure 2. Evolutionary relationships of the CARP4/SAARP1 sub-family of high acidic coral skeletal proteins indicate that these are coral-specific genes. The closest non-cnidarian blast hits, two *Crassostrea gigas* sequences, to the three groups -CARP4/SAARP1, CARP5/SAARP2, and P27/acidic SOMP/SAARP3 – exhibit blast E-values of -20 to -25. All gene names are the first letter of the genus name and first three or four letters of the species name plus protein

identification number from comparative.reefgenomics.org (Bhattacharya et al. 2016) or contain the NCBI accession number.

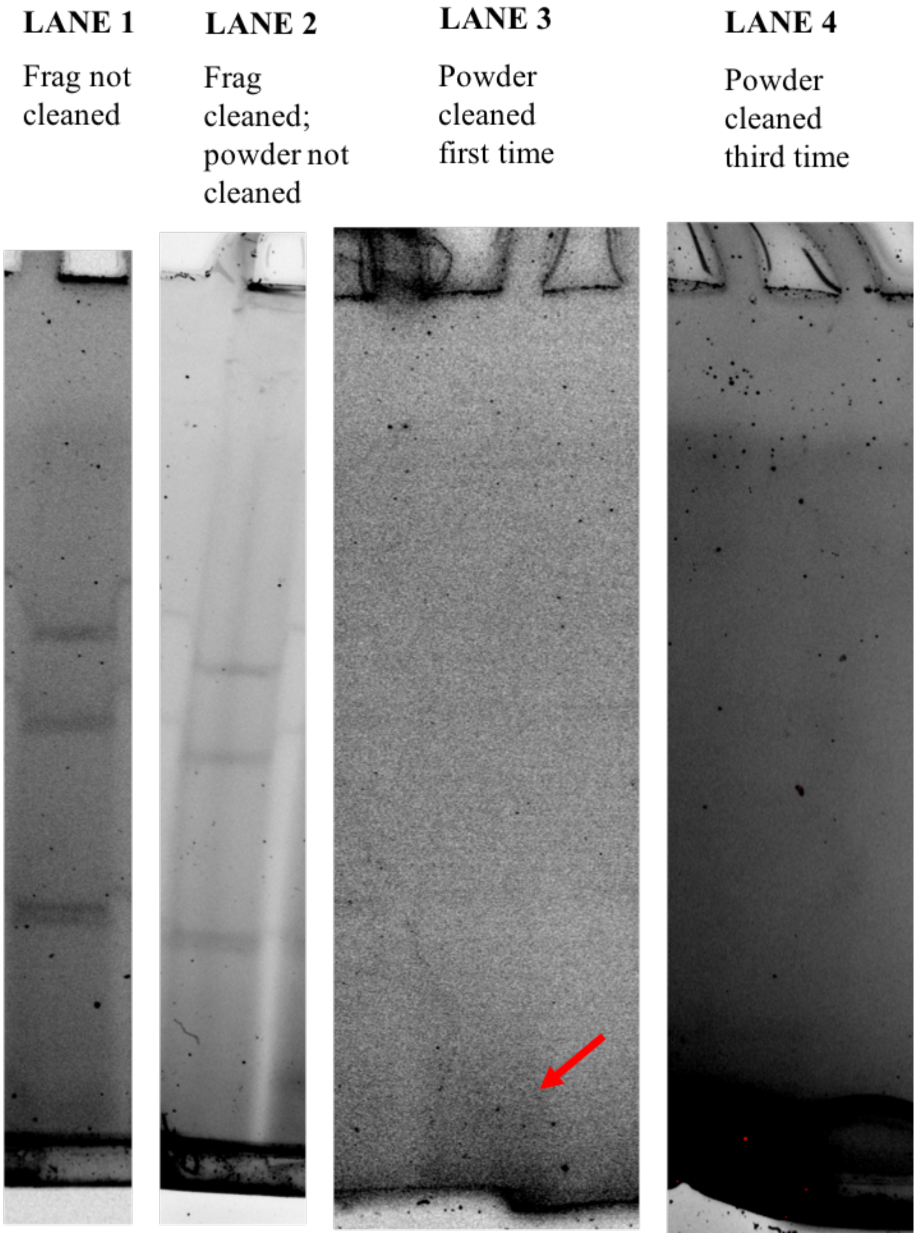


SI Figure 3. Concentrated PBS that had been soaked on modern *Porites lobata* skeleton fragments (frag) and powders and run on a 4-20% gel by SDS-PAGE. Modern coral sample cleaning is shown as they are more likely to have existent cellular contamination. A smear of proteins is clearly visible for PBS soaked on an uncleaned frag and on powder from a cleaned fragment. A faint smear (most visible in the low molecular weight peptides; red arrow) is also apparent in *P. lobata* powders cleaned one time. No proteins were detected by SDS-PAGE ($4^{th}$ lane) or BCA (SI Table 4), and minimal amino acids were detected by protein hydrolysis and amino acid analysis after cleaning the skeleton powders three times.

**SI Tables**

SI Table 1. Modern and fossil coral skeleton descriptions.

SI Table 2. Fossil coral protein and peptide sequences. Peptides detected by LC-MS/MS are in **bold**.

SI Table 3. Modern *Orbicella annularis* skeleton top-most 3 cm depth skeletal proteome. AIM and ASM fractions were digested first in trypsin and then in GluC using the same method applied to the well-preserved Pleistocene *O. annularis* specimen, Mann4.

SI Table 4. Quantification of contaminant proteins and amino acids. Slabbed coral specimens powders were soaked in filtered phosphate buffered (PBS) saline while sonicating and the PBS soak was concentrated on Amicon 3 kDa filter units. Proteins in the soak of uncleaned (column B) and cleaned (column C) slabs, and powders that underwent one (column D) and then two (column E) rounds of oxidation and rinsing were quantified by bichronoic acid (BCA) assay. Absorbances of reacted proteins at 560 nm were very similar to that of a filtered PBS blank soaked in a conical vial during the same time as the twice-cleaned powder (column F); however, we conducted a third round of oxidative cleaning. After this third cleaning round, we hydrolyzed proteins in a further PBS soak in 7 M HCl and quantified total hydrolysable amino acids, by methods described in the main text, in this final soak (column G). Concentrated PBS soaks from thrice-cleaned fossil and modern coral powders had <5 nmol/g skeleton total hydrolysable amino acids, which is three orders of magnitude lower than the amino acid content of skeleton powders (Tomiak, 2016 Geochimica et Cosmochimica Acta).

SI Table 5. Mascot potential contaminant analysis. Mascot output of all fossil coral skeletal protein LC-MS/MS data run against a contaminants database and UniProt bacteria, cyanobacteria, fungi, and human databases. No peptides identified to coral sequences were associated with bacteria or fungi potential contaminating proteins. Further a process blank run on the instruments at the same time as the fossil coral skeletal protein sequencing revealed no proteins identified as coral proteins where the detected peptides were not also identical to human homologs.

SI Table 6. Statistical comparison of the relative amino acid content of fossil versus modern coral skeletal protein.

SI Table 7. PRIDE proteomics data repository reviewer login information.

**References**

Bhattacharya, D. *et al.* Comparative genomics explains the evolutionary success of reef-forming corals. *eLife* **5**, e13288 (2016).

Tomiak, P., Andersen, M., Hendy, E., Potter, E., Johnson, K., & Penkman, K. E. H. (2016). The role of skeletal micro-architecture in diagenesis and dating of *Acropora palmata*. *Geochimica et Cosmochimica Acta, 183*, 153-175.
